# Navigating next-gen nutrition care using artificial intelligence-assisted dietary assessment tools—a scoping review of potential applications

**DOI:** 10.3389/fnut.2025.1518466

**Published:** 2025-01-23

**Authors:** Anuja Phalle, Devaki Gokhale

**Affiliations:** Department of Nutrition and Dietetics, Symbiosis School of Culinary Arts and Nutritional Sciences, Symbiosis International (Deemed) University, Pune, India

**Keywords:** artificial intelligence, dietary assessments, mobile applications, machine learning, tele-nutrition, food image analysis, wearables

## Abstract

**Introduction:**

Recent developments in Artificial Intelligence (AI) and Machine Learning (ML) technologies have opened new avenues for their applications in dietary assessments. Conventional dietary assessment methods are time-consuming, labor-driven, and have high recall bias. AI-assisted tools can be user-friendly and provide accurate dietary data. Hence, this review aimed to explore the applications of AI-assisted dietary assessment tools in real-world settings that could potentially enhance Next-Gen nutrition care delivery.

**Materials and methods:**

A total of 17,613 original, full-text articles using keywords such as “artificial intelligence OR food image analysis OR wearable devices AND dietary OR nutritional assessment,” published in English between January 2014 and September 2024 were extracted from Scopus, Web of Science, and PubMed databases. All studies exploring applications of AI-assisted dietary assessment tools with human participation were included; While methodological/developmental research and studies without human participants were excluded as this review specifically aimed to explore their applications in real-world scenarios for clinical purposes. In the final phase of screening, 66 articles were reviewed that matched our inclusion criteria and the review followed PRISMA-ScR reporting guidelines.

**Results:**

We observed that existing AI-assisted dietary assessment tools are integrated with mobile/web-based applications to provide a user-friendly interface. These tools can broadly be categorized as “Image-based” and “Motion sensor-based.” Image-based tools allow food recognition, classification, food volume/weight, and nutrient estimation whereas, Motion sensor-based tools help capture eating occasions through wrist movement, eating sounds, jaw motion & swallowing. These functionalities capture the dietary data regarding the type of food or beverage consumed, calorie intake, portion sizes, frequency of eating, and shared eating occasions as real-time data making it more accurate as against conventional dietary assessment methods. Dietary assessment tools integrated with AI and ML could estimate real-time energy and macronutrient intake in patients with chronic conditions such as obesity, diabetes, and dementia. Additionally, these tools are non-laborious, time-efficient, user-friendly, and provide fairly accurate data free from recall/reporting bias enabling clinicians to offer personalized nutrition.

**Conclusion:**

Therefore, integrating AI-based dietary assessment tools will help improve the quality of nutrition care and navigate next-gen nutrition care practices. More studies are required further to evaluate the efficacy and accuracy of these tools.

## 1 Introduction

Nutrition Care Process (NCP) transitioned from face-to-face to Tele-nutrition consultations following the COVID-19 pandemic (1, 2), which inherently motivated the scientific community to develop innovative, technology-based tools to facilitate healthcare professionals. With the advancements in technologies such as Artificial Intelligence (AI) and Machine Learning (ML), efforts have been directed toward extending their applications to the field of nutrition (3, 4). However, much of the emphasis has been given to investigating various techniques using machine learning (ML), and deep neural networks that are required for app development rather than to check its feasibility to enhance nutrition care practices in real-world settings.

Dietary assessment is the primary step of NCP, requiring the collection and analysis of data, including dietary information (5). It is crucial for accurate nutritional diagnosis, optimum nutrition delivery, and faster patient recovery. Various conventional dietary assessment tools including 24-h dietary recall, 3-day food diary, and food frequency questionnaires (FFQ) have been used extensively in nutrition practice (6). However, healthcare professionals such as dietitians and nutritionists face challenges in obtaining accurate data using these conventional tools. For e.g., conventional tools are time-consuming, and labor-intensive, with a higher recall bias. Further, patients forget or misreport the consumption of snacks, especially unhealthy foods (7).

AI-assisted dietary assessment tools are user-friendly and can provide objective and accurate data rather than subjective information from self-reported questionnaires. ML and deep neural networks are the backbones of most AI-assisted tools (8, 9). To make these tools user-friendly, they have been integrated with various devices including smartphones, and other wearable devices like smartwatches/fitness trackers. Increased technological dependence observed in today’s modern society can be leveraged for active health and nutritional monitoring. Moreover, trends and heightened behaviors related to food photography were observed among the population post COVID-19 pandemic (10, 11). This behavior could be strengthened to promote real-time tracking of patient’s eating patterns and behaviors primarily using AI-assisted dietary assessment tools.

The potential clinical significance of AI-assisted dietary assessment tools cannot be overemphasized. For e.g., close monitoring of food intake in hospitalized patients is paramount to prevent malnutrition which is often underdiagnosed in this group (12–14). This can enhance nutrition care and improve patient’s quality of life in hospitalized settings. Furthermore, clinical conditions like Type I or insulin-dependent Type II diabetes require careful recording, adjusting, and monitoring of carbohydrate intake to ensure better glycemic control. Invigilating the nutrition status and dietary patterns of notable segments of the population such as children, adolescents, young adults, females of reproductive age, and pregnant women is essential. Previously, potential applications of AI-assisted dietary assessment tools have been reviewed with emphasis on the underlying technologies and methodologies of these tools (9, 15, 16) rather than studying possible applications in real-world settings. Furthermore, there is a lack of knowledge about challenges and research gaps regarding applications of AI-assisted dietary assessment tools. Therefore, we aimed to review the existing literature for its scope and highlight the shortcomings of these AI-based dietary assessment tools. Specifically, this scoping review focuses on AI-assisted dietary assessment tools that can potentially help assess and objectively monitor the dietary intake of humans in real-world scenarios as well as their relevance in clinical conditions in enhancing Next-Gen nutrition care.

## 2 Methods

The authors performed an extensive literature search using Scopus, Web of Science, and PubMed databases to include articles published in the last 10 years (2014 to 4th September 2024). A total of 17,613 original, full-text articles published in English were extracted using the keywords “artificial intelligence OR food image analysis OR computer vision-assisted OR wearable devices AND dietary OR nutritional assessment.” All studies (Experimental, Proof-of-concept, Pilot/Validation, Cross-sectional, Randomized Controlled Trial, Observational, Comparative, Exploratory, and Qualitative studies) exploring applications of AI-assisted dietary assessment tools and human participation were included; While methodological/developmental research studies were excluded as this review specifically aimed to explore their applications in real-world clinical use. We did not restrict ourselves to specific outcomes of interest, as we aimed to cover the state-of-the-art literature. In phase I, all articles were screened for title and abstract considering the inclusion–exclusion criteria (see Supplementary Table 1). 568 full-text articles were screened in phase II, and 66 were eligible for this review. Further, the reference list of all articles and published reviews was checked for eligibility (Refer to Supplementary Figure S1 for flowchart). Both the reviewers independently reviewed the articles, and the disagreements were resolved through mutual discussion. The review followed PRISMA-ScR reporting guidelines (Refer to Supplementary Table 2) and findings from studies were synthesized using a narrative approach. The literature was appraised and collated for applicability, feasibility, accuracy, and benefits to critical segments of the population in a real-world scenario.

## 3 A brief overview of AI-assisted dietary assessment (DA) tools

**Figure 1:**
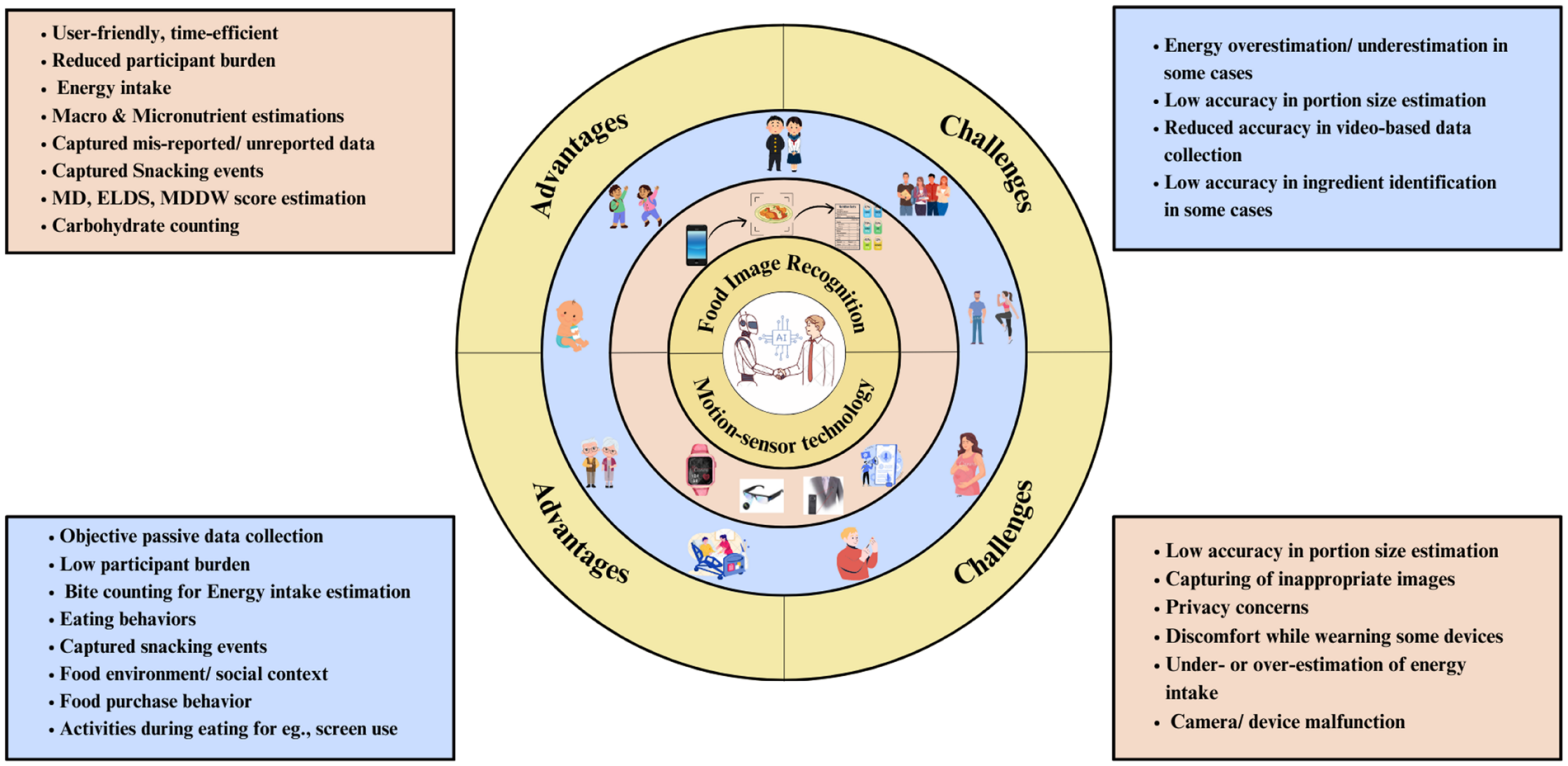
Overview of AI-assisted dietary assessment tools.

Advanced AI-assisted Dietary Assessment Tools are primarily based on technologies such as ML, Natural Language Processing (NLP), Deep Learning, Computer Vision, and Artificial Neural Networks including Convoluted Neural Networks (CNN). These technologies generally help in image classification, segmentation, recognition, and prediction (17). Current DA tools can broadly be classified as ‘Image-based’ and ‘Motion Sensor-based’ (See Figure 1) Food Image Recognition (FIR) is an extensively common feature of Image-based Dietary Assessment (IBDA) tools. IBDA tools are deployed through mobile/web applications, where the user snaps a picture of the meal through a mobile phone’s camera and gets the nutrients and volume estimation as an output. In between these steps, multiple other steps including image pre-processing, segmentation, food classification, volume estimation, and calculation of nutrients by establishing connections with appropriate nutritional databases (18). Another approach used in AI-assisted DA tools captures dietary data using sensor-based wearable devices. This technology enables the passive and objective method of obtaining dietary data. The wearable device detects a motion (hand, jaw motion, speech recognition) or captures images passively which are then processed further to provide output to the user. Various wearable devices such as e-buttons, smartwatches, and eyeglasses have been exploited in performing dietary assessments (19). Since this review explores potential applications in real-world settings in the human population, we present the existing literature based on population characteristics rather than a technology/feature-centered narrative.

### 3.1 For children & adolescents

Performing dietary assessments in children and adolescents is very challenging. Conventional dietary assessment tools capture dietary data from parents/caregivers leading to recall bias, misreporting, and under-reporting. However, very few studies have explored the challenges in dietary data reporting among children in the past with only two reviews highlighting the gap in this domain (20, 21). Adolescents tend to underreport more than young children, especially those with overweight and obesity. The issue of underreporting has been observed to reduce in young children when parents help them in data reporting, however, any subjective reporting can introduce recall or reporting bias. AI-assisted tools may help mitigate these challenges for this group providing more reliable and accurate data. We identified 12 eligible articles, of which 7 explored ‘IBDA’ and 5 implemented ‘Wearables’ to perform dietary assessments in children and adolescents (Table 1).

**Table 1:** AI-assisted dietary assessment tools for children and adolescents.

Image-based dietary assessment tools for children & adolescents								
Author & Year	Country	Study design	Tool used	Study population	Sample size	Objectives	Advantages	Disadvantages
Fialkowski MK et al., 2022	Hawaii	Cross-sectional study	Baby mFR	Infants (3–12 months) & Surrogate caregivers	70 infants & surrogate caregivers	Eating occasions of infants Adherence to app Willingness and desirability of the app’s features	Feasible, easier to use than writing food intake 91% adherence to food intake reporting 40% of caregivers reported breastfeeding events including timings of breastfeeding sessions (1–120 min) and number of days (Highest on weekends) Use of the app did not make caregivers conscious of type of feeds given to baby & did not lead to changes in foods fed	–
Wang JS et al., 2019	Taiwan	Pilot study	COFIT app	Children (6–17 years)	23	Feasibility of COFIT in nutrient estimation	Moderate correlation between nutrient estimations by COFIT & 3-FR Acceptable ICC Low bias as per Bland–Altman analysis	Significant differences in energy and fat estimations Average difference in energy intake was 194 kcal/day.
Johansson U et al., 2018	Sweden	Pilot Validation study	Image-based FR	Infants 12 months	22	Image-based FR vs. traditional 5-day FR Energy and nutrient estimations	No differences in EI & macronutrient estimation between both methods High reliability (ICC = 0.81)	10% overestimation of EI compared to total energy expenditure (TEE)
Marcano-Olivier M et al., 2019	UK	Validation study	Fixed FIR system	Children 5–18 years	239	FIR vs. traditional WFR in crowded school cafeteria	Children consumed over 80% of the provided food. Low bias and high accuracy (89.40%) Inter-rater agreement- good levels of inter-rater agreement (Cohen’s κ = 0.535–0.819)	Low accuracy for fruit & vegetable intake by 10.55%
Ptomey, LT et al., 2015	USA	Feasibility study	FIR	Adolescents with Intellectual & developmental disease	20	Nutrient Estimation & Feasibility	Participants were able to capture 68.3% of eating occasions	Overestimation of the energy, macronutrients, and micronutrients (*p* < 0.05)
Folson GK et al., 2023	Ghana	Cross-sectional	FRANI app	Adolescents (12–18 years)	36	Feasibility & accuracy of FRANI app	Acceptable CCC for nutrient estimation between FRANI and WR (0.30 and 0.68)	High (31%) omission rate for FRANI than WR 16% intrusion rate with FRANI compared to 24-h recall
Wearable sensor-based dietary assessment tools for children & adolescents								
Beltran A et al., 2018	USA	Cross-sectional Feasibility Study	Wearable, E- button	Children 9–13 years	30	Assessment of e-button camera for food identification Interrater validity by dietitians, child–parent interviews for food & caloric intake	Dietitians agreed that 60.5% of images were accurately captured by e- button Acceptable ICC (0.65) before & after verification interviews	Camera captured all the images irrespective of child’s intake for e.g., cup of coffee by parents Inability in portion size estimation (only 24% of foods got measured by e-button) Problem with food identification (12.4% of the foods were identified)
Zhou Q et al., 2019	China	Cross-sectional	Narrative Clip 2	Children	52	Narrative Clip 2 vs. 3-day, 24-h recall	Narrative Clip 2 captured underreported & misreported data Snacks- most commonly underreported (40%) followed by beverages (37%), fruits (30%), snacks, and desserts (16%)	Conventional dietary recall method underestimated energy intake by 149 ± 182 kcal/d (8%)
Raber M et al., 2018	USA	Observational study	e-Button	Adolescents aged 9	31	Food preparation behaviors & practices Intercoder reliability	Browsing the pantry/fridge was most common behavior and getting drinks High (89.1%) Inter-rater agreement, moderate/high inter-coder reliability (Cohen’s Kappa = 0.667). Most common food preparation work was unwrapping & combining 2–3 ingredients 51.6% observed food preparation by adults Microwaving was most common cooking method	–
Veatupu L et al., 2019	Tonga	RCT	Kidscam	Children 10–12 years	108	Assessment of eating behaviors, and patterns	High consumption of non-core foods (unhealthy, processed-ultra-processed foods, sugar-rich beverages, desserts, cookies, pastry) High snacking frequency Low fruit consumption Consumption of non-core foods was highest in school and while walking on the roads (outside of home) 97% of purchases were non-core foods	–
Jobarteh ML et al., 2023	UK	Feasibility study	Wearable	Kenyan & Ghanian children	17 children	Nutrient analysis & Portion size estimation	Eyeglasses and e-button had the highest acceptability High image clarity was seen with the e-button but the high quality of food visibility was seen with eye glasses Wearable devices had good ICC The Bland–Altman analysis showed a good degree of agreement with no significant bias between the two methods The ICC value was 0.75, showing a good degree of agreement (reliability) between the two methods for portion size estimation	underestimation of portion size by 14% Passive dietary assessment underreported macro and micronutrients
Kamar M et al., 2019	UK	Pilot Feasibility Study	Sensecam	Adolescents 11–16 years	8	Sensecam was used to check consumption of whole grains and factors influencing them	Low consumption of whole-grains Participants associated brown-colored food with rich in whole-grain Confusion, peer influence, and social context affected whole grain consumption	–

mFR, mobile food record; COFIT, Fit together; ICC, Intra-class correlation coefficient; EI, Energy Intake; FIR, Food image recognition; WR, Weighted Food Records; FRANI, Food Recognition Assessment & Nudging Insights; CCC, concordance correlation coefficient.

Considering the differences in the frameworks used in AI and ML models, the accuracy of AI-based tools may vary.

#### 3.1.1 Image-based dietary assessment

A cross-sectional study by Fialkowski et al. (22) assessed the feasibility and user-friendliness of the ‘baby mobile Food Recording (mFR) app by asking surrogate reporters like caregivers to record the eating occasions of 70 infants (3–12 months). A majority (94%) of surrogates recorded the infant’s dietary intake using the mFR app and 75% of the before-after images were visible. Only 40% of surrogates recorded breastfeeding events but the data gave insights into breastfeeding days and duration. Although breastfeeding events occurred every day, the duration ranged between 1 and 120 min with longer breastfeeding duration observed on weekends. Surrogates reported that the app was feasible and user-friendly and they would prefer taking food images rather than writing. Furthermore, using the app did not make surrogate reporters conscious about feeding compositions and did not change the infant’s feeding pattern, thus enabling the capturing of actual data. Another study from Sweden compared nutrient estimation (energy and macronutrient) using image-based food records (FR) with conventional food records for 5 days. Additionally, the study validated the image-based FR by estimating total energy expenditure (TEE) using doubly labeled water (DLW). Energy intake estimated by image-assisted FR (3,905 ± 476 kJ/day) was no different than conventional FR (3,902 ± 476 kJ/day) and had a high (0.81) intra-class correlation coefficient (ICC). This indicates high reliability with image-based FR. However, 10% of the overestimation of energy intake with image-based FR was observed compared to TEE estimated using DLW (23).

A pilot study was conducted among 23 Taiwanese children (6–17 years) to check the feasibility and accuracy of the COFIT (meaning fit together) image-based FR app against the conventional written FR. Energy intake estimated using COFIT (1,584.88 ± 369.03 kcal/day) was significantly (*p* ≤ 0.001) lower than written FR (1,779.11 ± 316.29 kcal/day). For other macronutrients (except fat) and micronutrient estimations with a moderate correlation (0.27–0.97) between both methods. Bland–Altman plots were within the acceptable limits of agreement, indicating small variability in nutrient estimation by the COFIT app (24). Similarly, the UK study assessed the validity and accuracy of the digital image-capture method to estimate the food consumption of 239 children (5–18 years) in a school café as opposed to conventional weighted records (WR). 80% of the food served for lunch and dinner at the café was consumed by children. The accuracy of food group estimation was similar with minor bias; however, significantly low accuracy (10.88%) was observed for the fruits & vegetable group (*t* = 2.893, *p* = 0.004). Nevertheless, overall good inter-rater agreement (Cohen’s κ = 0.535–0.819) indicated better reliability of the digital image capture method (25).

A cross-sectional study validated the FRANI (Food Recognition Assessment & Nudging Insights) app against conventional weighted records and multiple-pass 24-h dietary recall among 36 Ghanaian adolescents. The FRANI app was equally good at estimating energy intakes within a 10% equivalence bound like conventional weighted records and even better than 24-h recall, which was accurate at a 20% equivalence bound. Micronutrients such as folate, iron, zinc, niacin, and vitamin B6 were accurately estimated within 15% bounds by the FRANI app except for vitamin A and B12 which had wider equivalence bounds (>30%). The rates of omission (31%) and intrusion (16%) errors were higher concerning food consumption episodes for FRANI and WR compared to 24-h recall which had lower omission (21%) and intrusion rates (13%) (26). Another feasibility study explored whether digital images help improve energy and nutrient estimation compared to conventional 3-day food records (FR) among adolescents with Intellectual and Developmental Disorders (IDD). Participants could record 68.3 ± 31.7% of eating occasions. Digital images could detect incorrect portion size (37.4%), missing data (28.2%), forgotten food intake, and false positive intake (2.3%) which were not captured in conventional 3-day FR. Significant differences in intakes of energy, carbohydrate, protein, and fat were observed (*p* < 0.05 for all), wherein digital images captured a lot (20%) of under-reported, misreported data as opposed to 3-day FR. The study concluded that adolescents with IDD could easily capture food images and this method may be feasible for estimating their nutrient intake (27).

Overall, the image-based method was user-friendly and feasible for dietary assessment in infants, children, and adolescents including those with IDD. Capturing an infant’s true dietary intake including breastfeeding events (which is challenging otherwise) with an image-based app provided objective data collection. The nutrient estimation with the image-based method in children had minor variations, but within acceptable limits, indicating fair reliability. The dietary intake of adolescents is often a tricky task for healthcare professionals however, image-based apps appear to ease out their task by providing fairly accurate estimates of energy, macronutrients, and most micronutrients. Further, this method detected 20% of the misreported and unreported dietary data compared to conventional tools. Therefore, image-based dietary assessment tools may be next-gen tools for healthcare professionals.

#### 3.1.2 Wearable sensor-based dietary assessment

A wearable e-button device was evaluated for feasibility and accuracy for 2-days among 30 children against child–parent dyad interviews. E-button is a wearable button-shaped device with a mini camera that captures the food images every 4 s apart. This device accurately detected around 60.5% of images and had acceptable inter-class correlation agreement (0.65). Child–parent interviews by dietitians confirmed that 77% of foods were accurately identified by e-button, however, 12.4% of foods were not detected by e-button for incorrect camera position, dark settings, blurred images, and camera malfunctions. Furthermore, the e-button could not always detect the true food intake of a child leading to the capturing of false-positive images for instance, the child did not consume the food but helped in food preparation, gave cookies to a sibling, or happened to be sitting at a table where parent’s cup of coffee was kept. Concerning portion size, 67.8% of food portions were estimated using an e-button. Significant differences in energy intake were observed where dietitians underestimated energy, further child–parent dyad verification interviews revealed an energy intake of 287.8 kcal which was neither detected by e-button nor dietitians (28). A feasibility study from the UK investigated the validity of 3 different wearables (An e-button, an Automated Ingestion Monitor (AIM) eye-glasses device, and an ear-worn Bluetooth device with a mini camera) against traditional weighted food records among Ghanaian and Kenyan-origin children (*n* = 17). Children showed a higher preference for AIM devices than e-button. High image clarity was seen in the e-button however, the AIM device showed high visibility of the food plate. Wearable devices could fairly estimate portion size (*r* = 0.75, *p* = 0.01) and good (0.75) inter-class correlation coefficient (ICC) indicating high reliability, however, they underestimated the portion sizes by 14%. Concerning energy, macronutrients, and micronutrient estimation, wearable devices showed good ICC agreement values ranging between 0.67 and 0.91 (29). Similar findings were observed in a Chinese study among 52 children which assessed the feasibility of a Narrative Clip 2 wearable camera device worn for 7 days compared to a 3-day 24-h recall. Significant differences were observed in energy and nutrient estimations between both methods wherein 3-day 24-h dietary recall significantly underestimated energy, macronutrients, and micronutrients, however high correlations were observed between them (0.69–0.97, *p* < 0.001). Probably, because children often may not clearly remember the portion size the way they can remember what they eat when reporting 24-h dietary recall. This is where image-assisted dietary assessment tools can be advantageous in preventing the underreporting and misreporting of dietary data (30).

We found three studies that extended the applications of wearable devices to identify food behaviors, eating patterns, food environment, and whole-grain consumption rather than only exploring the feasibility and accuracy, thus providing different dimensions to its usage in real-world settings. An observational study among 31 American adolescents (9–13 years) captured food preparation and practices using e-Button showed a high inter-rater agreement (89.1%) and good inter-coder reliability (Cohen’s Kappa = 0.67). The ordinary food-related behaviors were checking in the fridge (71%) or pantry (51.6%), fetching the drinks (58.1%) and clearing the dishes (71%). The most common food preparation activities were mixing ingredients to prepare the dish (45.2%), opening the food packs (41.9%), watching adults cook (51.6%), and microwaving (22.6%). Overall, the e-button was effective in recording food-related behaviors and preparation activities, however, image processing required 2 h for processing. Nevertheless, the device was in the nascent stage, and being improvised further (31). A randomized control trial among 36 Tongan children investigated the pattern and context of traditional Tongan me’akai foods using Kids’Cam-a wearable camera device. The study reported the highest consumption of non-traditional foods (67.7%) twice more frequently (4.5 [95% CI 3.3, 6.7] times/day) than traditional Tongan foods (2.3 [95% CI 1.8, 2.9]). Importantly, a large portion of non-traditional foods were consumed as snacks mostly unhealthy (33.8%) such as cakes/cookies/desserts (21.6%) and confectionary items (25.6%). Additionally, identifying the contexts that promote unhealthy non-traditional foods/snack consumption is crucial in planning interventions and policy-making. This study explored the same wherein, unhealthy foods were commonly eaten at home (1.3 times), at school (3.5), and highest while walking outside on roads (5 times). Regarding food purchasing, children bought (97.7%) unhealthy foods as observed through images (32). Similarly, a pilot feasibility study assessed the contexts that can impact whole grain consumption among adolescents using SenseCam-a wearable camera. Most factors that prevented the consumption of whole grains were taste, availability at home, and outside the home in different settings, confusion regarding whole grains, and peer and social influence. Adolescents related brown-colored foods as whole grains and had negative taste perceptions of whole grains. They mostly ate whole grains (brown bread) at home rather than outside due to the greater availability of refined grains (white bread). Adolescents mostly related consumption of whole grain foods during diet interventions otherwise they feared being mocked for making odd choices in a social context (33). Using passive data collection methods wearables facilitate an in-depth understanding of various aspects that can significantly influence dietary intake which has never been possible with conventional dietary assessment tools.

In summary, wearable devices in children and adolescents can be viable options for understanding their eating behaviors, particularly snacking. However, these devices may underestimate the nutrients and portion sizes consumed especially when the food consumed is small in size or due to poor image quality and small. Additionally, capturing the true food intake of children was not always possible (28). Moreover, wearables were shown to be efficient in capturing a lot of other information rather than just food images, for e.g., food environments. Therefore, we conclude that wearables may be efficiently used for understanding different aspects of food rather than nutrient estimation until the accuracy is improved further.

### 3.2 For healthy and pregnant women

Womanhood is a challenging stage of life and requires optimal nutrition during each phase. Malnutrition (under and overnutrition) has become increasingly prevalent among women. Pregnancy is the crucial phase of a woman’s life wherein nutritional requirements increase substantially to meet the demands of a growing fetus. Past literature highlights the discrepancies in actual nutritional intake and recommended guidelines, indicating that most pregnant women do not meet the requirements. This issue is serious among pregnant women from low-middle-income countries (LMICs), therefore capturing their actual objective intake becomes of utmost importance in preventing negative consequences. Further adolescents and healthy women, like other adults, also tend to underreport or misreport during conventional dietary assessments. We narrate the findings from eight articles that explored the use of AI-assisted DA tools among women (Table 2).

**Table 2:** AI-assisted dietary assessment tools for healthy & pregnant women.

Image-based dietary assessment tools for healthy & pregnant women								
Author & Year	Country	Study design	Tool used	Study population	Sample size	Objectives	Advantages	Disadvantages
Braga BC et al., 2024	Vietnam	6-week pilot RCT	FRANI app	Female Adolescents (12–18 years)	36	Adherence to app Eating occasions Computation of diet quality scores	High adherence to gamified app (82%) 97% recording of meals Food consumption trends Higher scores DDS and the Eat Lancet Diet score in FRANI group	–
Nguyen PH et al., 2022	Vietnam	1-week pilot RCT	FRANI app	Female Adolescents (12–18 years)	36	Relative validity of FRANI vs. WR & multiple pass 24-h dietary recall	Good relative validity Accurate nutrient estimations	–
Serra M et al., 2023	Switzerland	Cross-sectional study	SNAQ app	Healthy female adults	30	1. Comparison between energy estimation by SNAQ app and DLW + 24 h dietary recall	SNAQ showed a slightly higher agreement (bias = −329.6 kcal/day) with DLW for total daily energy intake (TDEI) compared to 24HR (bias = −543.0 kcal/day). No significant differences in energy and macronutrient intake estimates between SNAQ and 24HR (Δ = 213.4 kcal/day).	Feasibility, user-friendliness, and participant satisfaction were not evaluated Compliance was not checked
McCloskey ML et al., 2019	USA	Qualitative study	Remote Food Photography Method	Mother–child dyad low-income, rural area	31	Number & quality of the photos received Participant feedback Meal timing Location Preparation & Quality	85% of the good-quality pictures were obtained App provided the opportunity to self-reflect on dietary patterns Mother and child both usually ate at the same time and the same food at home Low intake of fruits and whole grains Higher consumption of processed & ultra-processed	–
Ding Y et al., 2021	China	Feasibility study	FIR	Pregnant women	251 images, (Sample size of human participants was not mentioned)	WeChat-based (WAIDA) app for nutrient, portion size, and energy estimation	Compared with the weighing method, the variation range of food weight, energy, and nutrients estimated by the WAIDA method was smaller and more stable than that estimated by the recall method.	The Bland–Altman analysis showed the variability in energy and nutrient estimation
Ashman A.M et al., 2017	Australia	Feasibility study	FIR	Pregnant women	25	Feasibility Nutrient estimation Inter-rater variability	88% acceptability & willingness to use Acceptable Bland–Altman plot High ICC for all energy, macronutrients, and micronutrients	–
Wearable sensor-based dietary assessment tools for healthy & pregnant women								
Bulungu ALS et al., 2020	Uganda	Validation study	LLWC device	Mother & Child (13–23 months) dyads	211	Feasibility & validation of wearables for assessing DDS	Low relative bias for both 24HR (−0.1801) the Image-based method (−0.1227) The percentage of DDS that were identical comparing the Image-based or 24HR with the criterion method ranged was 58%	Both wearable and 24-h recall overestimated the diet quality
Bulungu ALS et al., 2023	Uganda	Cross- sectional study	LLWC device	Mothers with child between 13–23 months	211	Feasibility & user experience	Participants favored wearable device use Good feasibility was observed with wearable devices	Loss of 15% of data due to inoperability of the automated wearable camera

DDS, Dietary Diversity Scores; FRANI, Food Recognition Assessment & Nudging Insights; WR, Weighted Record; FIR, Food image recognition; WAIDA, WeChat Applet for Image-based Dietary Assessment; ICC, Intraclass Correlation Coefficient; LLWC, Life Logging Wearable Camera.

Considering the differences in the frameworks used in AI and ML models, the accuracy of AI-based tools may vary.

#### 3.2.1 Image-based dietary assessment

A randomized pilot study among Vietnamese and Ghanaian adolescent females (*n* = 36) assessed the feasibility of the FRANI (Food Recognition Assistance and Nudging Insight) app to capture their food choices and diet quality. This gamified app had high adherence (82%) and participants recorded 97% of the meals consumed. The study reported that most participants had low Minimum Dietary Diversity for Women (MDDW) and Eat Lancet Diversity Score (ELDS) meaning adolescent females did not consume most of the food groups (34). The same FRANI application was checked for relative validity by Nguyen et al. (35) against the weighted records and multi-pass 24-h recall. Energy, macronutrients, and micronutrient estimations were between 10 and 20% equivalence bounds using FRANI and weighted records showing a good correlation between both methods (concordance correlation coefficient [CCC]-0.60 and 0.81). However, a higher correlation (CCC-0.70–0.89) was observed for 24-h recall and weighted records. A cross-sectional study among 30 healthy females validated energy and macronutrient estimation by the SNAQ image recognition app with the gold standard Doubly Labeled Water (DLW) technique and 24-h dietary recall. SNAQ app and 24-h recall tended to underestimate the energy intake compared to DLW. However, total energy intake estimated using SNAQ had a higher agreement with DLW with a bias of −239.6 kcal/day compared to 24-h recall which had a larger bias (−543 kcal/day) (36).

Two studies evaluated the use of IBDA among pregnant women and those experiencing motherhood. A study among Australian pregnant women examined the relative validity of DietBytes to estimate energy and nutrient intake. No significant differences between energy, macronutrients, and micronutrient estimations were seen between DietBytes and 24-h recall, indicating acceptable Bland–Altman plots with minimum bias. Further, the high ICC (0.93, *p* < 0.001) for energy, and macronutrients (ICC-0.865–0.932, *p* < 0.05) was attended between dietitians for DietByte. Participants (88%) favored using DietBytes and 84% showed satisfaction with the app (37). Another study of pregnant Chinese women demonstrated the applicability of WAIDA (WeChat-based app for image-based dietary assessment) against the conventional weighted food records. Portion size estimations by WAIDA (0.825) were significantly (<0.001) correlated with the weighted food records (0.520). Furthermore, energy and nutrient estimations by WAIDA were closer to weighted records, indicating good accuracy (38).

In a qualitative study of 31 mother–child dyads, the feasibility of the Remote Food Photography Method was evaluated. Participants could send 85% of good-quality pictures that revealed meal times wherein mother and child usually had meals only a few minutes apart. 20% of the foods were takeaway items indicating consumption of convenience, processed, and ultra-processed foods. Further, most of the meals (47%) eaten by the mother were from disposable plates and takeaway containers (39).

#### 3.2.2 Wearable sensor-based dietary assessment

Only two studies used wearable cameras to assess their feasibility and validity among mothers with infants. A cross-sectional study from Uganda, evaluated the validity of Life-logging wearable cameras (LLWC) to calculate Diet Diversity Scores (DDS), thus measuring maternal and child (*n* = 211 pairs) diet quality against weighted food records and conventional 24-h recall. The mean diet diversity (MDD) scores of mothers were higher when calculated using LLWC (42.3%) and 24-h recall (41.1%) as compared to weighted food records (47.2%). Both the methods, LLWC and 24-h recall had a low relative bias of −0.1227. Although the LLWC method showed low relative bias, it demonstrated moderate reliability (Cohen’s κ coefficient-0.59) compared to 24-h recall which had higher reliability (Cohen’s κ coefficient-0.68). Therefore, LLWC and 24-h recall could accurately estimate the DDS, however, both methods overestimated the scores more than direct weighted food records (40). Further the same group of researchers in 2023, examined the feasibility of LLWC devices in rural Eastern Uganda in collecting the dietary data of 211 mothers. Most participants rated LLWC devices as very good (56%) and good (36%). Importantly, 29% of participants reported LLWC as the less preferred method for reasons such as privacy invasion, fear associated with the device, and emotional burden in handling other’s reactions to device use. However, these results were not significantly higher than those who rated other methods as least preferred. This study also reported the technical challenges observed by researchers. For e.g., 15% data loss was a drawback of this method owing to inoperability, camera malfunction, forgetting to wear the device, and accidental video recording by the device (41).

Overall, IBDA and passive wearable camera devices were equally good, even slightly better than conventional methods in estimating portion sizes, energy, macronutrients, and micronutrients. The other findings from these studies showed that AI-assisted dietary assessment tools could calculate the diet quality scores, and analyze the meal times, meal composition, and intakes of unhealthy foods among adolescents, pregnant women, and mothers from LMICs. We conclude that these objective, passive data collection methods for dietary assessment were feasible and fairly accurate for use in this group.

### 3.3 For healthy adults

We observed that the applicability of AI-assisted dietary assessment tools was extensively studied in healthy, young-middle-aged adults than any other age group/health condition. There have been significantly more challenges in obtaining dietary data from young adults (18–35 years). For e.g., young adults tend to underreport and misreport snacking events (42–44). AI-assisted dietary assessments may be beneficial in capturing true information regarding unhealthy eating behaviors and patterns among adults (Table 3).

**Table 3:** AI-assisted dietary assessment tools for healthy adults.

Image-based dietary assessment tools for healthy adults								
Author & Year	Country	Study design	Tool used	Study population	Sample size	Objectives	Advantages	Disadvantages
Larke JA et al., 2023	USA	3-days RCT	FIR	Healthy adults (18–65 years)	95	Ingredient prediction using publicly available databases	–	Publicly available databases showed poor performance with failure to detect a single ingredient food items
Lucassen DA et al., 2021	Netherland	Evaluation study	FIR	Adults (20–70 years)	40	Image-based vs. text-based portion size	–	Portion size estimation using TB-PSE had better performance Bland–Altman plots indicated a higher agreement for TB-PSE
Ji Y et al., 2020	Canada	2 weeks, RCT	Keenoa	Healthy individuals 18+ years	72	Validity of Keenoa- participant, Keenoa- dietitian app against 3-day food diary	User-friendly app	Significant Underestimation of macro and micronutrient
Radtke MD et al., 2022	USA	Prospective cohort study	Diet ID app vs. NDSR-24	University (18+ years)	42	Carotenoid content 24-h recall micronutrient estimation Healthy Eating Index calculation	Accurate nutrient intake estimation Diet Diet quality assessed by HEI- was significantly correlated with Diet ID & NDSR	–
Roux de Bézieux H et al., 2021	USA	6-week, double-blind, placebo- controlled, 2 × 2 cross-over pilot study	FIR with CGM sensors	Adults (>17 years)	6	Food intake integrated with CGM devices	CGM integrated with FIR provided greater insights into meal-induced glucose peaks and compliance with diet regimen Study aimed at targeting reducing participant burden and recall bias Provides Visual diet log	–
Naaman R et al., 2021	UK	Comparative study	FIR (FP and VR)	Adults (>18 years)	84	Feasibility Nutrient estimation Inter-rater variability	Inter-rater reliability was strong for both IBDA methods in estimating energy intake (ρ-coefficients: FP = 0.80; VR = 0.81) Inter-class agreement of IBDA methods was moderate	FP (−13.3%) and VR (−4.5%) underestimated energy intake
Papathanail I et al., 2023	Switzerland	Feasibility study	FIR	Adults	50	Feasibility Nutrient prediction	The binary segmentation network achieved an intersection over union (IoU) of 74%, Low mean absolute percentage error in kcal estimation (27.41%).	Higher variance in calorie estimation compared to dietitians Higher percentage of errors for macronutrients (31.27% for the CHO, 39.17% for the protein, and 43.24% for the fat) estimation compared to the dietitians’ estimations
Harray, AJ et al., 2017	Australia	Cross-sectional	FIR	Young adults (18–30 years)	246	Intake of junk food & sugar-sweetened beverages	High intake of Junk food & sugar-sweetened beverages Gender differences were observed in junk food consumption	–
Chan KS et al., 2020	Malaysia	Cross-sectional	FIR	Young adults	46	Nutrient estimation Feasibility	Participants (67.4%) preferred IBFR method The Bland–Altman analysis demonstrated a good level of agreement between IBFR and 24DR for energy	–
Ziesemer K et al., 2020	Germany	Cross-sectional	FIR	Adults	183	Checking misreported data	38% of misreported events were due to individual factors	The most common reason for missing events was due to technical issues with FIR
Papathanail, I et al., 2022	USA	Feasibility study	FIR	Adults	24	Adherence to the Mediterranean diet	No significant differences in MD scores calculated by app and dietitian Accuracy = 57.3%	–
Kong NA et al., 2023	Malaysia	Qualitative study	FIR	Adults	30	Feasibility of app	High usability & acceptability scores usability	–
Qiu J et al., 2021	UK	Feasibility study	Video-based food recognition	Adults	12	Bites & food recognition using videography	High Accuracy = 74.15%	Video-based food recognition of consumed food items is challenging
Tufano M et al., 2024	Netherland	Feasibility study	Video-based food recognition	Adults	15	Bite counting using videos	Good accuracy with (79%) and without annotation (71.4%) in counting bites	–
Wearable sensor-based dietary assessment tools for clinical conditions								
Wang L et al., 2022	Australia	Cross-sectional	Wearable camera	Young adults (18–30 years)	41	Variations in eating occasions Identification of irregular dietary patterns	High variabilities between 1st meal and last meal were seen Young adults had irregular eating patterns	–
Gemming L et al., 2015	New Zealand	Validation study	Wearable camera	Adults	40	Validity of FIR against DLW for energy estimation	Underreporting was significantly reduced with a combined use of FIR & 24-h recall compared to multiple pass 24-h recall alone (*p* < 0.001). Identified snacking events	Gender differences concerning energy intake estimation were observed wherein both methods underestimated energy intake
Chou T et al., 2024	USA	Exploratory study	Smartwatch Bite counter	Adults	82	Estimation of energy intake and energy density	–	High variability (41.5%) in energy intake estimation
Lorenzon i G et al., 2019	USA	Cross-sectional study	Wrist band-bite counter	Young adults (20–36 years)	18	Wrist motion intake and correlation with energy intake	–	Overestimation of energy intake by 200 kcal Accuracy in energy intake estimation varied with type and amount of macronutrients present in food regardless of number of bites recorded.
Doulah A et al., 2020	USA	Cross-sectional observational study	AIM-2 Eyeglasses s	Adults	30	Feasibility & accuracy of active vs. passive food image capture Privacy concerns	High accuracy (82.7%) Active Image capture method had low privacy concern scores	The continuous capture method had high privacy concern scores (5.0 ± 1.6)
Pan ZX et al., 2022	USA	Cross-sectional study	Wearable camera	Adults	18	Energy estimation	ICC improved by 0.39% with a new method Bland–Altman analysis indicated strongly improved agreement between nutritionists.	–
Farooq, M et al., 2016	USA	Feasibility study	Wearable eyeglasses s	Adults	10	Food intake and physical activity detection	High average F1-score of 99.85%, High precision (99.89%), and better recall 99.82% of the two-stage classification method Eyeglasses could effectively identify the food intake	–
Gemmin g, L et al., 2015	New Zealand	Feasibility study	Sensecam	Adults	40	Assessment of environment, location, sitting positions, social interaction, screen usage while eating, social interaction	High inter-rater reliability Sensecam efficiently captured majority of snacking events The majority of eating episodes while standing/active were snacks (73%) and were energy-dense (2.9 kJ/g 95% 1.7 to 4.8) High screen use was seen irrespective of meal type Energy-dense snacks were consumed while viewing screens	–
Pettitt C et al., 2015	UK	Pilot study	Bluetooth earpiece	University (18+ years)	6	Energy intake	–	Under-reporting of energy intake rate of 34% compared to DLW.
Taylor S et al., 2021	USA	Pilot study	Speech sensor based COCO app	Adults	35	Energy and nutrient estimations by AI-based nutritionist COCO app vs. 24-h recall	No significant difference in energy intake by COCO app and 24-h recall (2,092 ± 1,044 kcal versus 2,030 ± 687 kcal, *p* = 0.70). No differences in macronutrients estimated by the COCO app	–
Chan V et al., 2021	Australia	Observational study	Wearable camera	Young adults (18–30 years)	133	Assessing the intakes of omitted foods comparing wearables, ASA-24, & App	A total of 1,822 eating occasions (main meal or snack) were identified using the wearable cameras	Snacks were more likely to be omitted in both the 24 h recall (*p* < 0.001) and app (*p* < 0.001) Water, beverages, sauces, and condiments were frequently omitted in traditional methods
Shen Y et al., 2017	USA	Experiment al study	Smartwatch	Healthy adults (18–70 years)	271	Accuracy of wrist motion for counting bites	High sensitivity (75%) for automatic bite detection	Chopsticks had low sensitivity Faster eating speed decreased the sensitivity by 3%
Chan V et al., 2022	Australia	Cross-sectional study	Wearable device	Young adults (18–30 years)	133	Energy density of meals & snacks consumed Preparation location and context- screen time, socialization	Snacks were more energy-dense than meals Home-cooked meals were lower in energy density Screen use was associated with food intake	Wearable device could not capture 360 eating episodes reported in 24-h recall
Dimitrat os SM et al., 2020	USA	Feasibility study	Wearable, Wristband	Healthy adults (18–50 years)	25	Feasibility of wristband to record the nutritional intake	High correlation between wearable devices and conventional dietary recall	Bland–Altman analysis showed a mean bias of −105 (SD 660) kcal/day, Wristband underestimated the higher caloric intake and overestimated the lower caloric intake Challenges in accurate estimation
Alshuraf a N et al., 2021	USA	Observation al study	Wearable camera	Adults (18–65 years)	16	Eating behaviors- eating speed, number of bites taken, and eating time of the day Energy intake	High energy intakes were associated with a higher number of bites, reduced eating speed, and high BMI Interestingly, reduced eating speed was associated with increased calorie intake	Identification of only 24% of eating episodes
Scott JL et al., 2024	Ireland	Exploratory study	Wearable cameras	Healthy adults (18–65 years)	20	Accuracy Nutrient estimation Capturing of unreported dietary data	Macronutrient estimations were higher Recorded 44 unreported items and 44 misreported food items Detection of processed food intake at snacks Participants reported the camera to be easy to use	Invasion of privacy while using bathroom, banks Participants became conscious of eating could lead to a depiction of normal food intake Camera could only provide idea about food pattern with no information regarding portion size

FIR, Food image recognition; TB-PSE, Text-based portion size estimation; NDSR, National Data System for Research Database software; IBDA, Image-based dietary assessment; FP, Food photography; VR, Video recording; IBFR, Image based Food record; DLW, Doubly labelled water; AIM, Automated Intake Monitoring.

Considering the differences in the frameworks used in AI and ML models, the accuracy of AI-based tools may vary.

#### 3.3.1 Image-based dietary assessment

We summarized the findings from 17 studies that investigated the use of IBDA in healthy adults. A recent randomized controlled trial among 95 adults was conducted to compare the feasibility of the SNAPMe (Surveying Nutrient Assessment with Photographs of Meals) app against conventional automated self-administered (ASA24) food records. 67% of participants preferred using the SNAPMe app while 85% confirmed that it captured their true intake. Participant burden was significantly low in both methods according to 78% of the participants. Regarding the food ingredients, SNAPMe showed poor performance in identifying single ingredients. Further, its integration with publicly available databases remained inefficient underscoring the significance of developing a high-quality, large database (45).

A study from the Netherlands evaluated the efficacy of portion size estimation using image-based or text-based methods and ASA-24. Portion sizes estimated using image-based methods for all foods and drinks varied significantly from true intake (46). In terms of estimating nutrients, studies presented mixed findings wherein few IBDA apps were better than others. In a recent study, the Diet ID app was validated with 24-h dietary recall. The energy, macronutrients and micronutrients estimated using Diet ID were significantly correlated with 24-h recall. Furthermore, the study also aimed to check the efficacy of Diet ID in estimating carotenoid intake. Diet ID carotenoid estimations were as reliable as plasma & skin carotenoid concentrations (47). Similar findings were observed with the image-based food records (IBFR) app. In this study, the nutrient estimations using the IBFR app were compared with conventional 24-h recall. The Bland–Altman plot showed good agreement and a strong correlation between both methods. Further 67.4% of participants favored the IBFR method over 24-h recall (48). In contrast, a randomized controlled trial by Ji et al. (49), reported the difference in nutrient estimations with the Keenoa-image recognition app and a 3-day food diary (3DFD). The intake of energy, macronutrients, and micronutrients differed significantly from 3DFD with Bland–Altman plots showing poor agreements for these crucial nutrients. Nevertheless, the Keenoa app was perceived as user-friendly by more than half (58.2%) of the participants.

The majority of the studies showed acceptable to strong inter-rater agreements meaning high accuracy and low variations in recognizing true foods or nutrient estimations despite few shortcomings (50, 51). A study by Naaman et al. (50) investigated the efficiency of IBDA methods such as food photography and video recording against weighted food records in 84 healthy adults. The study reported high inter-rater reliability for image-based (0.80) and video-recording-based (0.81) energy estimation. However, image-based (−13.3%) and video-recording (−4.5%) methods underestimated energy intake. Compared to weighted food records, IBDA methods were user-friendly, time-efficient, practical, and enjoyable (*p* < 0.05 for all). In another feasibility study, the goFOOD application was assessed for feasibility and accuracy. 69% liked using this app and 83.3% of them found it to be user-friendly. A higher accuracy of image segmentation (food recognition) (IoU-74%) was observed with goFOOD. Moreover, the app achieved a lower mean absolute error (27.41%) in energy intake calculations, but the error percentages were higher for all macronutrients (31–43%). Additionally, the goFOOD app overestimated the energy and nutrients (51).

Two studies checked the efficacy of image and video-based apps in counting bites consumed by participants. A study by Qiu et al. (52), comparing photo-based and video-based estimation of bites consumed, showed video-based method can accurately count the bites up to 74.15%. Additionally, the study concluded that recognizing visible foods is easier than detecting consumed food items resulting in a 25% decrease in accuracy levels. Similarly, in another study, counting bites from videography was 79% accurate with annotation and 71.4% without annotation (53). Only one qualitative study was conducted in this population which reported that IBDA apps were more acceptable, user-friendly, and feasible to use among this population (54).

Few studies showed the feasibility of IBDA for assessments of different dietary behaviors. For e.g., an Australian study using the IBDA app revealed a higher intake of junk food and sugar-sweetened beverages among young adults (3.7 ± 2.0) servings per day (55). Another cross-sectional study among adults aimed to perform Ecological Momentary Analysis (EMA) using image-based dietary assessment methods. In the EMA approach, the study of food environment, and social context are studied rather than only focusing on an individual’s food intake, therefore providing insights into factors that trigger certain eating behaviors. The study found underreporting of the snacks was a common missing data and actual context has a lot to do with eating behaviors (56). Another feasibility study examined the utility of the IBDA app in calculating Mediterranean diet (MD) scores against those calculated by dietitians. In this study, the mean precision of 57.3% was achieved with the IBDA app. Additionally, this food recognition system precisely calculated MD scores, whereas self-administered 24-h recall overestimated the MD scores (57).

Only one study attempted to integrate an image recognition app with data obtained from a Continuous Glucose Monitoring (CGM) device. The study concluded that integrating CGM devices with IBDA enlightened individuals about meal-induced glucose spikes, and adherence to dietary intervention. Additionally, it provided a visual representation of food intake and relative glucose monitoring data. Such breakthrough technological innovations can be highly effective and encouraging for individuals and healthcare providers (58).

#### 3.3.2 Wearable sensor-based dietary assessment

A total of 16 explored the feasibility of integrating wearable-sensor-based dietary assessment tools among healthy adults. A study by Gemming et al. (59) validated SenseCam—a wearable camera device and 24-h dietary recall against the DLW technique. This study reported a high underestimation of energy intake in men (17%) and women (13%) when measured only using SenseCam as opposed to SenseCam plus 24-h recall which was observed to reduce the underestimations (men %, women %). The proportion of underreporting in men (8%) and women (6%) was reduced significantly in SenseCam plus 24-h recall than the conventional 24-h recall alone (*p* < 0.001). Additionally, SenseCam could identify the unreported snacks (265 foods). The study concluded that using advanced AI-assisted DA in combination with conventional tools enhances data accuracy.

Three studies recorded the food intake using wearable eyeglasses (60, 61). A study by Doulah et al. (60), validated a unique AIM-2 (Automatic Ingestion Monitor) wearable eyeglasses to detect eating events without invading the user’s privacy. AIM-2 device had a high accuracy (82.7%) in detecting eating events. Furthermore, the study showed high privacy concerns scores for continuous capture (5.0 ± 1.6) versus image-capturing (1.9 ± 1.7) only during eating events on a 7-point Likert scale. Another study using wearable eyeglasses assessed the novel functionality of capturing the food consumed during physical activity. The study showed two-stage classification system was better (F-1 score-99.85%) and had higher precision (99.89%) and recall (99.82%) than the single classification. This means that wearable eyeglasses could accurately predict eating events during physical activity (61). Another cross-sectional study using the AIM-2 wearable device showed an improved ICC by 0.39 in agreement with the Bland–Altman analysis (62).

Wearable devices often help capture eating behaviors and occasions. A study from the USA provided wearable cameras to 16 adults to record the association between the number of bites, eating speed, and energy intake. The study concluded that reduced eating speed and a high number of bites consumed correlated with high BMI and energy intake. Interestingly, a reduction in eating speed does not necessarily mean low energy intake as slow eating speed was observed mainly with screen use. Despite these observations, wearable cameras could identify only 24% of eating events (63). Another study counted bites with the help of a smartwatch. This method had good sensitivity (75%) and a prediction rate of 89%. However, sensitivity was significantly reduced by faster eating speed and chopstick use (64). A cross-sectional study from Australia among young adults explored intra-individual differences in eating timings using a wearable camera device for 3 days. The results indicated high variabilities in their eating events and patterns, indicating irregular dietary patterns among adults (65).

About five studies exploring nutrient estimation through different wearable devices, presented mixed findings. A wrist-band bite counter device overestimated the energy intake varied with the amount & type of macronutrient present regardless of the number of bites recorded (66). Another study combining the two technologies such as the bite counter with IBDA, reported higher variability in estimations of energy intake with the bite counter (41.5%) compared to IBDA (23.4%) (67). Similar findings were observed in the UK study investigating the wearable Bluetooth earpiece against the DLW method to estimate energy intake among adults. The energy intake was underestimated by almost 34% when calculated using a wearable Bluetooth earpiece (68). A feasibility study from the USA reported that wearable wristbands significantly underestimated high-calorie and overestimated low-calorie intake with a bias of −150 ± 660 kcal/day as per Bland–Altman plots. The study reported challenges in accuracy and high variability with wristbands (69). In contrast, a speech-assisted (using the NLP model) app (nutritionist COCO) estimated energy and macronutrients, similar to that obtained from 24-h recall (70).

Snacking is common among young adults leading to increased energy consumption, mostly associated with higher screen usage. The Australian cross-sectional study showed the same using wearable cameras, where snacks had higher energy density than home-cooked meals and snacking episodes were significantly associated with screen use. Contrary to the notion, this study reported that wearable cameras could not capture 360 eating events reported in 24-h recall (71). Further, wearable cameras overestimated macronutrients in some cases, but were found to be useful in capturing 44 unreported and misreported items. Additionally, wearable cameras were efficient in the detection of processed food intake during snacking without any bias. However, the study noted a few downsides to this approach as it may lead to an invasion of privacy and make participants conscious of eating thus affecting the capture of true intake (72).

### 3.4 For hospitalized patients and clinical conditions

Malnutrition is often underdiagnosed in critically ill hospitalized patients and greatly affects disease prognosis, length of stay, and overall quality of life (12–14). Critical assessment and monitoring of food intake in critically ill/hospitalized patients is paramount. Performing dietary assessments in hospitalized patients and those with clinical conditions has been challenging wherein the conventional tools fail to capture the objective and true dietary data. AI-assisted dietary assessment tools may potentially benefit these vulnerable populations and provide accurate information (Tables 4, 5).

**Table 4:** AI-assisted dietary assessment tools for clinical conditions.

Image-based dietary assessment tools for clinical conditions								
Author & Year	Country	Study design	Tool used	Study population	Sample size	Objectives	Advantages	Disadvantages
Moyen A et al., 2022	Canada	2 weeks Randomized cross-over design	Keenoa app	Healthy Individuals + individuals with diabetes	136	Validity of Keenoa app against ASA-24	High usability ratings for Keenoa app Accurate prediction of macronutrients and few micronutrients	–
Borges T.L.D et al., 2023	Brazil	Cross-sectional observational study	FIR	Individuals with Visual impairment	40	Food identification through photos taken by individuals with visual impairment Nutrient estimation	Healthcare professionals were able to estimate food consumption patterns through pictures taken by individuals with visual impairment, identifying 11/13 images	Correct estimation of food amount for 23% of dishes only Nutrient estimations were underestimated by photography method than WFR
Kerr D.A et al., 2017	New Zeal	RCT	mFR	Young adults with obesity (18–30 years)	212	Willingness to capture food images was assessed	High participant willingness to use mFR than WFR High willingness and adherence to app was seen in participants with high BMI	–
Heikkilä L et al., 2021	Finland	Comparative study	FIR	Type 1 DM	13	Feasibility Nutrient estimation Carbohydrate estimation	Moderate to excellent in EI estimation (ICC = 0.91, *p* < 0.001) Good to excellent in carbohydrate intake estimation (ICC = 0.95, *p* < 0.001). The mean carbohydrate intake did not differ between the methods	Young people tend to forget to take pictures The image-based method underestimated the mean energy intake by 5.5% (mean difference-88 ± 131 kcal/day, *p* = 0.033).
Rhyner D et al., 2016	Switzerland	Comparative study	goCarb	Type 1 DM adults	19	Carbohydrate estimation User experience Usability	Significantly (*p* = 0.001) Low mean absolute error for the GoCARB system was (12.28 ± 9.56) grams of carbohydrate In 75.4% (86/114) of the meals, the GoCARB automatic segmentation was successful 85.1% (291/342) of individual food items were identified	–
Makhsous S ET AL., 2020	USA	Exploratory study	FIR	Individuals with diabetes	12	Accuracy of Dietsensor for individuals with diabetes	The lowest average absolute error on the collected data was for Dietsensor (33%) compared to 24-h (73%), & MyFitnessPal (51%) app	–
Vasiloglou MF et al., 2018	Switzerland	2 months, comparative study	goCarb app	Dietitians	6	Carb counting using goCarb diet vs. dietitians Accuracy of visual estimation by goCarb & dietitians with weighted method	The lower mean absolute for goCarb system (14.8 ± 9.73) & Dietitians (14.9 ± 10.12) grams of carbohydrates Accuracy of GoCarb was 37.03%, vs. 35.2% dietitians Significant correlation between ground truth and Gocarb (*p* < 0.001)	The size of the food affected carbohydrate estimation by both GoCarb and dietitians
Wearable sensor-based dietary assessment tools for clinical conditions								
Murphy J et al., 2017	UK	Cross-sectional study	Sensewear at arm	Adults with dementia in 2 care homes	20	Energy intake	Accurate energy intake estimation Significant correlation with DLW	–
Turner- McGrievy GM et al., 2017	USA	6-month, RCT	FIR *Vs* Wearable bite counter device	18–65 years old adults with Overweight/obesity	81, 42 in the FIR app group & 39 in the Wearable group	Adherence to weight loss intervention was checked by App vs. wearable device vs. traditional self-monitoring	Significant weight change was observed in the app group compared to the bite counter device Weight loss at 6th month was significantly correlated with a number of days intake was recorded (*r* = −0.33, *p* < 0.01) Both groups lost weight at 6th month compared to the baseline	–

ASA-24, Automated Self-Administered 24 h Dietary Recall; mFR, Mobile food records; WFR, Weighted Food Records; ICC, Intraclass Correlation Coefficient; EI, Energy Intake; DLW, Doubly labeled Water.

Considering the differences in the frameworks used in AI and ML models, the accuracy of AI-based tools may vary.

**Table 5:** AI-assisted dietary assessment tools for hospitalized patients.

Image-based dietary assessment tools for hospitalized patients								
Author & Year	Country	Study design	Tool used	Study population	Sample size	Objectives	Advantages	Disadvantages
Papathanail I et al., 2021	Switzerland	Pilot study	FIR through RGB camera operated by trained personnel	Hospitalized older adults	28	Comparison of before and after meal consumption to estimate intake Food segmentation performance Comparison between the system and 24-h recall	Specifically, it achieved a mean Intersection over Union (IoU) of 73.7%, a mean accuracy of 84.1%	High cost for camera set-up
Wymelbeke- Delannoy VV et al., 2022	France	Cross-sectional study	FIR	Hospitalized patients	20	Food Recognition Estimation of portion size	149 dishes were evaluated for before & after consumption Reliability was 39% for 58 dishes only	Low accuracy for portion size estimation
Honda M et al., 2022	Japan	Single center prospective study	FIR	Patients aged 32–72 years, undergone radical gastrectomy for GI cancer	10	Evaluation of meal pictures analyzed by health care providers on systems	94.6% of the images taken were evaluable Estimation of food & calorie intake	–
Ofei, KT et al., 2019	Denmark	Pilot study	DIMS 2.0	Hospitalized patients	17	Evaluation of DIMS 2.0 vs. Conventional WFM	No statistically significant difference in energy intake estimated by DIMS 2.0 and the WFM A high correlation between the DIMS and WFM was found. High ICC for portion size estimates of each food item before & after (*p* < 0.01)	–
Ryu J et al., 2024	South Korea	Single center prospective study	RGBD camera	Hospitalized patients from the Nephrology & Urology department	25	Sodium intake evaluation using FIR with the help of RGB camera, before & after images	RGBD-assisted sodium values may be considered as indicators for sodium intake with variations based on diuretics	Significantly (*p* = 0.02) inaccurate estimations (RGBD-2,022.7 mg) in AI-Na (adjusted) vs. Urinary-2,783.0 mg

ICC, Intraclass Correlation Coefficient; RGBD, Red Green Blue Display; DIMS 2.0, Dietary Information Monitoring System; WFM, Weighted Food Method.

Considering the differences in the frameworks used in AI and ML models, the accuracy of AI-based tools may vary.

#### 3.4.1 Image-based dietary assessment

We found four studies that tested the performance, accuracy, and nutrient estimation capacity of IDBA among hospitalized patients. Each of these studies concluded that IDBA may be used effectively in hospital settings in the future by addressing the existing challenges in methodologies. A pilot study from Denmark showed that Dietary Intake Monitoring System (DIMS) 2.0 effectively calculated energy intake when compared to the conventional method without systemic bias. Further DIMS 2.0 computed portion size before (0.88) and after (0.99) consumption with significantly high ICC agreement (*p* < 0.01) (73).

Similar findings were noted among 28 hospitalized older adults by Papathanail et al. (74) where the food image recognition system had a high mean accuracy of 84.1% and system surpassed the trained staff in nutrient estimation. In contrast, the IBDA system showed low reliability (39%) for around 58/149 dishes checked for portion-size estimation before & after (75). A single-center feasibility trial was conducted among hospitalized patients who underwent GI surgery to estimate portion size and nutrient intake. Around 94.6% of the images were evaluated by a food recognition system and provided fair estimates of food and nutrient intake (76). A South Korean single-centered prospective study explored the application of an AI-based dietary sodium detection system. The study reported significant differences in sodium estimations by AI-based and 24-h urinary sodium and concluded that sodium intake is affected by various factors thereby leading to inaccurate estimations by AI-based system (77).

Dietary assessments among individuals with diabetes are of prime significance and can help analyze their dietary pattern, adherence, and even estimation of carbohydrate intake which becomes important specifically for individuals with Type I diabetes and those taking insulin. We observed five studies conducted using IBDA apps among individuals with diabetes. A 2-week randomized controlled trial showed that the Keenoa app had high usability ratings with an accurate prediction of all macronutrients. However, the accuracy was high for only a few micronutrients (78). Similarly, another exploratory study from the US reported that DietSensor accurately estimated nutrient intakes of individuals with diabetes with lower absolute error (33%) compared to conventional 24-h recall (73%) (79). On the other hand, a comparative study from Finland reported that IBDA significantly underestimated energy intake by 5.5% (*p* = 0.033). Nevertheless, IBDA efficiently computed carbohydrate intake with an excellent level of agreement (ICC = 0.95, *p* < 0.001) (80). Equivalently, GoCARB- an image-based carbohydrate-counting app showed a remarkable performance (*p* = 0.001) with the lowest mean absolute error of 12.28 ± 9.56 g compared to conventional tools (27.89 ± 38.20). Moreover, GoCARB recognized about 85.1% of food items accurately (81). In another study by Vasiloglou et al. (82), comparing GoCARB versus experienced dietitians reported consistent findings wherein the GoCARB system (mean absolute error- 14.8 ± 9.73) matched the carbohydrate estimation to that of experienced dietitians (14.9 ± 10.12). The accuracy of GoCARB was 37.03%, almost outperforming dietitians (35.2%). In a study among individuals with overweight/obesity, the results indicated a higher willingness (OR-1.68, 95% CI-1.02–2.77) to capture images for more days (>14 days) rather than written records (83). Only one study evaluated the application of IBDA among individuals with visual impairment. Unlike previous findings, this system frequently underestimated the nutrients and estimated the correct food amounts only for 23% of items. Nevertheless, it was observed that healthcare professionals successfully identified food consumption (11/13 food items) from images captured by the individuals with visual impairment (84).

#### 3.4.2 Wearable sensor-based dietary assessment

A Wearable Sensewear armband worn by older adults with dementia was used to investigate whether arm movements can be correlated with energy intake. This study showed promising results wherein energy intake significantly (*p* < 0.05) correlates with total energy expenditure (85). Another 6-month randomized controlled trial assessed and monitored the adherence to diet intervention and calculated weight loss through a food recognition app and a wearable bite counter device. A significant correlation (*r* = −0.33, *p* < 0.01) was observed between weight and the frequency of intake recording (number of days). In conclusion, measuring and monitoring dietary intake significantly improved adherence to intervention leading to greater weight loss among individuals with overweight/obesity (86).

### 3.5 Other studies

Two studies exploring the utility of IBDA and wearable devices conducted in different populations are reviewed here. An observational study evaluated the eating behaviors of 20 families using a wearable smartwatch. Overall, the compliance rate was 89.3% for all the captured eating movements and 76.5% of movements were true positives with a precision of 0.77 (87). Another mixed-methods study among dietitians who used voice and image-assisted apps reported that capturing snacks and recipe recording was challenging. Additionally, dietitians experienced discomfort in capturing food photos in social settings (88) (Table 6).

**Table 6:** AI-assisted dietary assessment tools (Studies among other populations).

Author & Year	Country	Study design	Feature	Study population	Sample size	Objectives	Advantages of applied AI-based tool	Disadvantages of applied AI-based tool
Bell B.M et al., 2022	USA	Observational study	Wearables- Smartwatch	20 families	58	Eating behaviors	The overall compliance rate was 89.26% 76.5% (302/395) of the detected events were true eating events (i.e., true positive)	–
Saronga N et al., 2021	Tanzania	Mixed methods study	Voice and image-assisted app	Dietitians	18	Feasibility	Easy to use, acceptable by dietitians	Challenges such as capturing snacks, and discomfort in taking photos in front of others were observed.

## 4 Strengths and limitations of AI-assisted dietary assessment tools

We observed a developmental trend with AI-assisted dietary assessment tools in recent years following the COVID-19 pandemic. AI-assisted dietary assessment tools were user-friendly, time-efficient, feasible for usage in real-world settings, and highly favored by human participants. Most of the image-based and sensor-based (wearables) tools had accuracy ranging between 60 and 95%. Energy macronutrient and micronutrient estimations were as precise as those with conventional tools such as 24-h dietary recall. In some cases, it was even more precise than conventional tools when validated against gold-standard methods like DLW. Both image-based and sensor-based assessments captured more data than just the food intake and nutrient estimations. For e.g., food environments, social context, peer influence, shopping behavior, physical activities during eating, eating behaviors, and snacking events. AI-based dietary assessments could successfully identify misreported and unreported food intake. Few studies demonstrated the use of AI-based dietary assessments integrated to calculate dietary scores such as Mediterranean diet scores, Eat-Lancet Diversity Scores, and Minimum Dietary Diversity for Women. Furthermore, we described the potential applications of AI-based tools for vulnerable populations. Although these tools were beneficial, no technology is without limitations. The portion size estimations using wearable devices were inaccurate, wherein small portion sizes were consistently overestimated. Few AI-assisted tools either underestimated or overestimated the energy requirements. Technical malfunctions of the wearable devices caused the loss of data and some devices were more convenient to use than others. Importantly, privacy is the major concern for passive, automated, wearable devices that capture images continuously. Few studies reported capturing pictures other than participants’ food intake (for e.g., use of lavatory). Only one study developed a method of eating event-specific capturing of data ensuring high privacy to participants. It must be noted that each of these studies had specific objectives and were developed using different machine-learning models and frameworks which can significantly affect the performance of these AI-assisted tools. In other words, the lack of uniformity in the underlying frameworks of the apps and wearables may affect our conclusion making it difficult to generalize the findings. A few inherent limitations of this study must be acknowledged while interpreting the results. We could not assess the quality and risk of bias considering the lack of uniformity in research settings, design, study samples, and the evaluated outcomes in these studies. Nevertheless, our study highlights the research gaps while presenting the current state of evidence. It is worth noting that these technologies are still evolving, and more rigorous scientific efforts can be directed at testing the applications of these AI-based tools in real-world clinical settings.

## 5 Conclusion

In summary, various Image-based and Motion sensor-based AI-assisted dietary assessment tools exist with wider functionalities such as food identification, classification, calorie estimation, eating frequencies, and shared eating occasions. Such high-end functionalities can be efficiently used in hospitals, clinics, and tele-nutrition practices. Furthermore, AI-assisted dietary assessment tools are user-friendly, time-efficient, and can facilitate early nutrition intervention leading to rapid patient recovery. Therefore, integrating AI-based dietary assessment tools will not only help improve the quality of nutrition care but also navigate next-gen nutrition care practices. More studies are required to further evaluate the efficacy and accuracy of these tools. Additionally, we recommend training healthcare professionals in the optimum utilization of AI-assisted dietary assessment technologies to upgrade their clinical practices.
